# Reversible cerebral vasoconstriction syndrome following red blood cells transfusion: a case series of 7 patients

**DOI:** 10.1186/s13023-015-0268-z

**Published:** 2015-04-22

**Authors:** Hui Liang, Ziqi Xu, Zhijun Zheng, Haiyan Lou, Wei Yue

**Affiliations:** Department of Neurology, The First Affiliated Hospital, School of Medicine, Zhejiang University, Hangzhou, China; Department of Neurology, Hengdian hospital of Zhejiang Province, Zhejiang, China; Department of Radiology, the First Affiliated Hospital, School of Medicine, Zhejiang University, Zhejiang, China; Department of Neurology, Tianjin Huanhu Hospital, qixiangtai road 122, 300060 Tianjin, China

**Keywords:** Reversible cerebral vasoconstriction syndrome, Blood transfusion, Chronic anemia

## Abstract

**Background:**

Reversible cerebral vasoconstriction syndrome (RCVS) is an infrequent disease characterized by severe headaches with or without focal neurological deficits or seizures and a reversible vasoconstriction of cerebral arteries. The Orpha number for RCVS is ORPHA284388. However, RCVS triggered by blood transfusion is rare. Here we provided the clinical, neuroimaging and outcome data of patients diagnosed with RCVS resulting from red blood cells transfusion.

**Methods:**

We retrospectively identified 7 patients presenting with RCVS after red blood cells transfusion from January 2010 to May 2014. The information on clinical features, neuroimaging and outcome were collected and analyzed.

**Results:**

All 7 patients were Chinese women, with a mean age of 42 years (38–46). All the patients had severe anemia (Hb level < 6 g/dl) caused by primary menorrhagia due to uterine myoma (n = 5) or end-stage renal disease (n = 2) and severe anemia persisted for a average period of 4 months (2–6). Each patient received packed red blood cells transfusion (average: 1580 ml) over a period of 2–5 days. Blood transfusion increased the hemoglobin level by at least 4.5 g/dL from baseline. The neurological symptoms appeared a mean of 6.3 days (2–13) after the last blood transfusion. Headache was the most frequent symptom and seizure, transient or persistent neurological disorders were observed. Neuroimaging showed cortical subarachnoid hemorrhage (n = 2), focal intracerebral hemorrhage (n = 2), localized brain edema (n = 3), cerebral infarction (n = 1), and posterior reversible encephalopathy syndrome (n = 2). Cerebral vasoconstrictions were demonstrated by magnetic resonance angiography or cerebral angiography. Arterial constriction reversed in all patients within 1 to 3 months of follow-up after disease onset and no relapse was observed up to a mean of 17.1 ± 4.8 months of follow-up.

**Conclusions:**

RCVS is a rare complication as a result of blood transfusion in patients with chronic severe anemia and should be considered in patients who show severe headache or neurologic deficits after transfusion.

## Background

Reversible cerebral vasoconstriction syndrome (RCVS) is an infrequent disorder characterized by severe headaches with or without focal neurological deficits or seizures and a reversible segmental and multifocal vasoconstriction of cerebral arteries [1]. The most common clinical feature of RCVS is severe acute headache, often presenting as a thunderclap headache [2,3]. The major complication of RCVS is ischemic or hemorrhagic stroke, which may lead to transient or permanent symptoms. RCVS occurs predominantly in females with ages close to 50 years [2]. The exact pathophysiology of RCVS remains unknown and the prevailing hypothesis favors an involvement of transient disturbance in the control of cerebral vascular tone [4]. RCVS may occur spontaneously or be provoked by various factors. The most common factors are postpartum and exposure to various vasoactive substances, including illicit drugs and selective serotonin-reuptake inhibitors [2,3]. Blood transfusion is a commonly used therapy for chronic severe anemia, but it has not been considered as an etiology of RCVS. So far only few cases have been reported [5,6].

In this report, we characterized the clinical features, neuroimaging and outcome of patients diagnosed with RCVS resulting from red blood cells transfusion.

## Subjects and Methods

Patients who developed RCVS after receiving red blood cells transfusion were retrospectively identified from January 2010 to May 2014. The study was approved by the hospital’s research ethics committee and written consent was retrospectively obtained from patients and their legal representatives.

The diagnosis of RCVS after blood transfusion was based on the following criteria [2,7]:(1) acute and severe headache (often thunderclap) with or without focal deficits or seizures; (2) segmental vasoconstriction of cerebral arteries confirmed by indirect (eg, magnetic resonance or CT) or direct catheter angiography; (3) complete or substantial normalization of arteries within 12 weeks after clinical onset;(4) history of receiving red blood cells transfusion because of severe anemia within 3 months. Seven patients were diagnosed in the study based on imaging or neurological deficits. Among them, 3 were from the emergency, 2 from neurology outpatient clinic and the rest 2 from gynecology. Other potential precipitating factors of RCVS, such as vasoactive substance use, post-partum status and prior immunosuppressant use, were excluded. In addition, all the patients did not have medical or surgical conditions associated with RCVS, including neurosurgical procedures, head trauma, catecholamine-secreting tumors, and carotid endarterectomy.

All patients underwent neuroimaging evaluations within 7 days after admission. Neuroimaging examinations included cerebral computed tomography (CT) scan (n = 7); cerebral 1.5 or 3.0 Tesla MRI (n = 7) with diffusion weighted-images (DWI), fluid-attenuated inversion recovery (FLAIR) images, T1WI, and T2WI; two-dimensional time-of-flight cerebral MR angiography (MRA) and magnetic resonance venography (n = 5); cervical and transcranial doppler ultrasonography (TCD) (n = 6) and cerebral angiography (n = 2). The scans were conducted and analyzed independently by a neuroradiologist and a neurologist.

Laboratory data were analyzed for all patients within 72 hours after admission, including routine blood tests, liver and kidney function, C-reactive-protein level, erythrocyte sedimentation rate, antiphospholipid antibodies. Lumbar puncture was performed in 3 patients.

The data were collected through review of the medical charts and interview with the patients and/or proxy, including: 1) sex, 2) age, 3) history of primary headache disorders, hypertension, pregnancy, and delivery, 4) reasons of anemia and total volume of red blood cell transfusion, 5) medications and drugs taken before the onset of the disorder, 6) detailed characteristics of present headaches, blood-pressure, and neurological symptoms, and 7) treatment and follow-up visits.

## Results

All 7 patients were Chinese women, averaging 42 years (38–46). The demographic characteristics of patients are listed in Table 1. No patients had a history of hypertension. The causes of severe anemia were primary menorrhagia due to uterine myoma (n = 5) or end-stage renal disease (n = 2). The duration of severe anemia (Hb level < 6 g/dl) lasted for an average of 4 months (2–6). The hemoglobin before transfusions was 2.6 g/dl (1.5-3.4). Each patient received packed red blood cells (PRBCs) transfusion (average: 1580 ml (750–2500)) over a period of 2–5 days. The patient’s hemoglobin increased to an average of 10.2 g/dL (7.9-12.5) after transfusion. Two patients received an operation of total abdominal hysterectomy on the second day after blood transfusion and one patient received 400 mL PRBCs after surgery. Propofol, sufentanil and rocuronium were used for anesthesia. Dezocine was administered and they only described mild incisional pain.

**Table 1:** **Demographics and clinical data in 7 patients with RCVS following red blood cells transfusion**

**Patients**	**Sex/age (years)**	**Cause of anemia**	**Duration of anemia (Hb level < 6 g/dl) (month)**	**BT volume of PRBCs (ml)/days**	**Hgb (g/dl) before/after BT**	**Symptoms after last BT (days)**	**Symptoms**	**Brain lesions on CT/MRI scans**	**Vascular imaging**
1	F/46	Menometrorrhagia (uterine myoma)	2	1700/4	3.3/11.6	6	Mild headache, hemiplegia (L)	cSAH	constriction of MCA
2	F/38	Menometrorrhagia (uterine myoma)	4	1800/4	2/12.5	2	Thunderclap headache	cSAH,	Diffuse distal constriction of MCA and ACA
3	F/45	End stage renal disease	5	800/2	3.4/7.9	4	Thunderclap headache, transient blurred version	Bilateral parietooccipital white matter FLAIR hyper-intensities, infarction	constriction of left posterior cerebral artery
4	F/40	Menometrorrhagia (uterine myoma)	6	2400/5	1.5/10.2	13	Thunderclap headache, dysarthria, hemiplegia (R)	bilaterall parietal edema, bilateral intracranial hemorrhage	segmental narrowings of bilateral ACA, MCA (L)
5	F/47	End stage renal disease	5	750/3	3/10.4	7	Thunderclap headache, transient blurred version, GTCS	Bilateral occipital white matter FLAIR hyper-intensities	constriction of MCA and PCA
6	F/39	Menometrorrhagia (uterine myoma)	2	2500/5	2.4/11	5	Thunderclap headache, GTCS,	slight cortical and subcortical edema (L)	constriction of MCA
7	F/44	Menometrorrhagia (uterine myoma)	4	1150/4	2.5/8.1	7	Mild headache, hemiplegia (L), mild cognitive impairment,focal motor seizure evoving into GTCS	Intracranial hemorrhage, localized subcortical edema (R)	Diffuse proximal and distal constriction of MCA and ACA

ACA, anterior cerebral artery BT, blood transfusion; cSAH, cortical subarachnoid hemorrhage;F, female; FLAIR, fluid-attenuated inversion recovery; GTCS, generalized tonic-clonic seizure; Hgb, hemoglobin; MCA, middle cerebral artery; PCA, posterior cerebral artery; PRBC, packed red blood cell.

### Neurological symptoms and signs

The neurological symptoms occurred in patients 6.3 days (2–13) after the last blood transfusion. Headache was the most frequent symptom in all 7 patients, of which 2 with mild headache and the other 5 with multiple thunderclap headaches. All episodes of thunderclap headaches occurred when patients were at rest. Pain location was bilateral in 3 patients. The maximal pain intensity was graded as a mean of 9 on the verbal scale. Recurrent thunderclap headaches occurred in 4 patients and the duration of severe pain was about 60 min (45–70). Two patients who had a history of migraine claimed that their headaches were completely different from that of their usual occurrence. Three patients had higher blood pressure during their acute headache and none of the elevation of blood pressure was persistent.

Five patients displayed other neurological symptoms. Focal deficits were seen in 5 patients and seizures occurred in 3 patients. In addition, 2 patients had only transient neurological symptoms, while 3 had a persistent deficit. Moreover, 3 patients showed neurological symptoms 1 to 4 days after headache onset, while other 2 had thunderclap headache concomitant to focal deficit. Hemiparesis, visual symptoms, dysarthria were common symptoms. One patient had cognitive impairment (mini-mental status examination [MMSE] score: 20). Among 3 patients displaying seizures, 2 had generalized tonic– clonic seizures (GTCS) and the rest had focal motor seizure turning into GTCS.

### Blood and cerebrospinal fluid analysis

The biochemical test showed elevated blood urea nitrogen and serum creatinine in 2 patients with the end-stage renal disease. CSF test were abnormal in 2 patients: 1 had slightly elevated CSF WBC (10/mm^3^) and the other had slightly elevated CSF protein level (49 mg/l). Both 2 patients showed elevated intracranial pressures (220 and 195 mmH_2_O respectively).

### Neuroimaging

**Figure 1:**
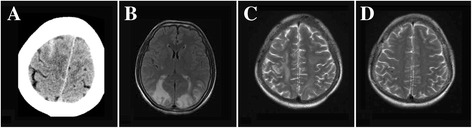
Brain imaging in RCVS. **(A)** CT scan showing cSAH; **(B)** FLAIR showing bilateral hypersignals in the occipital lobes consistent with PRES; **(C,D)** MRI showing right hyperintense subcortical lesions consistent with vasogenic edema and right brain hemorrhage;and cranial MRI showing resolution of the lesion after 12 weeks from the same patient as shown in **C**.

In all patients, CT scan was the first investigation. The initial CT was scanned at a mean of 1.3 days after headache onset, showing a cortical subarachnoid hemorrhage (cSAH) in 2 patients and parenchymal hemorrhage in another 2patients (Figure 1).

MRI scan images were apparently abnormal for all patients. MRI showed a small localized unilateral cortical subarachnoid hemorrhage (n = 2), localized brain edema (n = 3), focal intracerebral hemorrhage (n = 2) and cerebral infarction (n = 1). Two patients had MRI FLAIR hypersignals consistent with PRES on the first MRI performed at 5 days after headache onset (Figure 1).

**Figure 2:**
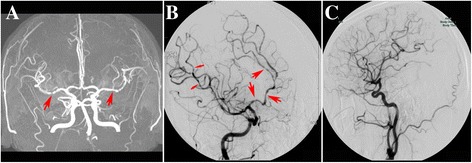
Vascular imaging in RCVS. **(A)** MRA showing the multiple narrowings of bilateral middle cerebral artery (red arrow); **(B)** cerebral angiography showing multiple narrowing and dilatations of middle cerebral artery (thin red arrow) and anterior cerebral artery (thick red arrow); **(C)** follow up angiography showing resolution of cerebral vasoconstriction after treatment from the same patient.

MRA showed diffuse segmental arterial constriction in 5 patients (Figure 2). Among 7 patients who had at least one TCD, 5 had increased arterial velocities with a mean of 157 ± 20 cm/s on middle cerebral arteries and 145 ± 14 cm/s on carotid siphons. Two patients had a conventional 4-vessel angiography that showed multifocal segmental arterial constriction (Figure 2). No unruptured aneurysms, vertebral or carotid artery dissection and venous sinus thrombosis were found in the study.

### Treatment

Nimodipine was used in 3 patients by initial intravenous infusion (1–2 mg/h adapted to blood pressure levels) followed by oral administration. The other 4 patients received only oral administration of nimodipine (30 mg, 3 times a day, adapted to blood-pressure levels). The duration for the treatment was ranged from 4 to 8 weeks. Antiepileptic treatments were used for all patients showing seizure activity. It was noted that recurrent headache, seizure and new neurological deficits occurred in 1 patient.

### Follow-up and clinical outcome

Follow-up study demonstrated the reversibility of arterial constriction in all patients within 1–3 months after disease onset, with a normal MRA (n = 5) or conventional angiography (n = 1). All patients showed complete resolution of the lesions. We observed that 1 patient showed a decreased maximum mean flow velocity in the middle cerebral arteries by the TCD test 30 days after onset and that the vasoconstriction recovered 1 year after by DSA examination.

The mean duration of follow-up was 17.1 ± 4.8 months. All the patients had a resolution of severe headaches and did not develop any other new neurological deficits 3–6 weeks after discharge. No recurrent RCVS or thunderclap headache occurred during the rest of follow-up. Two patients received total abdominal hysterectomy.

## Discussion

Several complications are associated with transfusion of blood products, many of which can be grouped as immunological or infectious [8]. However, neurological complications resulting from the blood transfusion has been rarely reported. The present series of 7 consecutive patients reported here is the largest group of RVCS caused by PRBC transfusion. The diagnostic criteria in the study allowed us to rule out other disorders responsible for headache and cerebral arterial constrictions such as primary or secondary cerebral angiitis or vasospam complicating an aneurismal subarachnoid hemorrhage. . None of the drugs used in anaesthesia of 2 patients has known sympathomimetic effects, which could be explained as vasospasm [9].

All the cases in the study were exclusively female patients at the middle ages with chronic severe anemia (5 patients suffered menorrhagia and 2 patients had an end stage renal disease). They all received PRBCs transfusions (average: 1580 mL) over a period of 2–5 days. Blood transfusion increased the hemoglobin level by at least 4.5 g/dL from baseline (average increase: 7.6 g/dL). Braun et al. reported previously that patient may present with symptomatic RCVS 3 months after transfusion [7]. However, in our cases, the neurological symptoms appeared an average of 6.3 days after the last blood transfusion. The discrepancy between the observations between our study and others [7] is still not clear.

Our results further confirmed that thunderclap headache is the clinical hallmark of RCVS [2]. These hyper-acute and excruciating headaches were immediately recognized by the difference from their previous headache in the patients who had a history of migraine. Some patients with RCVS may present mild headache [2]. For instance, two patients showed mild headache in our study. Different degrees of vasospasm in different vascular beds in the central nervous system may be responsible for the varying severity of headaches in different patients.

In addition to headache, focal neurological deficits of varying severity and seizures were frequent symptoms. cSAH occurred in 2 patients, while focal intracerebral hemorrhages were found in another 2 patients. They occurred early in the course of RCVS and were revealed mostly by a persisting focal deficit concomitant with thunderclap headache. Three patients were found with local brain edemas on MRI images and 2 patients had MRI FLAIR hypersignals, which were consistent with PRES. In our study, only 1 patient showed infarction at the surrounding regions in PRES.

There have been no reports of RCVS that occurs as a result of transfused blood products under conditions of acute blood loss. It has been hypothesized that chronic anemia may be accompanied by cerebral vasodilatation to compensate for ischemic hypoxia [10]. A rapid increase in the hemoglobin level and viscosity during blood transfusion might result in a loss of vasodilation and increase in vascular resistances, which cause ultimately overwhelming cerebral vessel constriction. This resembled to a cerebral hyperperfusion syndrome after carotid endarterectomy or stenting in carotid stenosis [11,12]. Immunologic and biochemical factors, including endothelin-1, catecholamines, and nitric oxide, might play a role in the pathophysiology of vasoconstriction in RCVS [13]. The latencies from autoregulatory breakthrough to symptom onset and vasoconstriction of major cerebral arteries may depend on the patients’ conditions, underlying diseases, amount and duration of blood transfusion [8,9,14]. Another factor for the delayed onset of vasoconstriction might be related to the temporal course of centripetal progression of the vasoconstrictions. The disturbance in the control of cerebral arterial tone might first involve small distal arteries, which were beyond the imaging studies [2]. Injury related to reperfusion may lead to the release of free radicals and trigger dysfunction of the endothelium, which may further aggravate cerebral vasoconstriction and damage to the vascular endothelial system, resulting in a vicious cycle. There have been reported that analgesics or emotion might trigger RCVS. In the study, dezocine was administered to alleviated pain for 2 total abdominal hysterectomy patients.

Therefore, we couldn’t exclude the possibility that analgesic or emotional distress potentially cause sympathetic activation and contribute to RCVS [15].

The occurrence of PRES has already been reported in RVCS [16]. PRES and RCVS share many clinicoradiographic features, including similar predisposing factors, clinical presentations, and clinical courses, suggesting that they may have similar pathophysiological mechanisms. It was possible that endothelial dysfunction after blood transfusion affected the regulation of cerebral arterial tone and triggered vasoconstriction with subsequent hypoperfusion, breakdown of the blood–brain barrier, and vasogenic oedema.

Although the use of nimodipine is controversial in the treatment of RCVS, it seemed to be effective in our study. The favorable response to nimodipine might be partly due to its role in preventing vasospasm or its effect on the endothelium [17]. Consistent with the previous literatures [1], clinical outcome of our patients was globally well.

According to previous reports, females were predominantly susceptible to RCVS. Also, most cases of PRES after blood transfusion that have been described in the literature were Asian patients [5]. We couldn’t exclude the possibility that there was an ethnic difference in the occurrence of blood transfusion associated RCVS.

## Conclusion

In conclusion, our cases presented in this report suggested that RCVS was one of the complications resulting from the blood transfusion in patients with chronic severe anemia, in particular, in women with menorrhagia. RCVS should be considered in patients if they developed a severe headache or neurologic deficit after transfusion.

## Abbreviations

cSAH: Cortical subarachnoid hemorrhage
CSF: Cerebrospinal fluid
MMSE: Mini-mental status examination
PRBCs: Packed red blood cells
PRES: Posterior reversible encephalopathy syndrome
RCVS: Reversible cerebral vasoconstriction syndrome
